# The British Journals: Materia Medica

**Published:** 1839-01

**Authors:** 


### MATERIA MEDICA.

### *Counter-Irritant Lotions.* By Dr. Granville.

[The following are the formulæ referred to in the note to p. 73 of the present Number of our Journal.]

Each kind of lotion consists of three ingredients:

1st. *The strongest liquor of ammonia*, A;

2d. *Distilled spirit of rosemary*, B;

3d. *Spirit of camphor*, C.

A. Saturate a given quantity of distilled water, contained in a glass receiver surrounded by ice, with ammoniacal gas, obtained in the usual way, from a mixture of equal parts of hydrochlorate of ammonia and recently slaked lime, both reduced to a fine powder. The water may be made to take up nearly 800 times its bulk of ammoniated gas under the circumstances described; its specific gravity will then be about 872, and 100 parts of it will contain 33 parts of real ammonia according to Sir H. Davy's tables. This solution of ammonia will, therefore, be more than three times the strength of the liquor ammoniæ of the Pharmacopœia of London, 100 parts of which, at a specific gravity of 960, contains only ten parts of real ammonia. I have, therefore, called mine "liquor ammoniæ fortissimus."

B. Take two pounds of the tips or small leaves of fresh rosemary, and eight pints of alcohol; leave the whole in infusion for twenty-four hours in a well-covered vessel, and after adding a sufficient quantity of water as will just prevent the empyreumatic smell, distil over seven pints. The Pharmacopœia of London directs the essential oil of rosemary to be distilled instead with rectified spirit. Such a preparation I found unsuited for my purpose.

C. To four ounces of pure camphor add two pints of alcohol, so as to dissolve the camphor, which solution should be filtered. The present tincture of camphor of the Pharmacopœia of London contains one ounce more of that substance, and does not harmonize so well with my two other ingredients as the weaker preparation.

The three ingredients, thus prepared, every medical man should keep always ready at hand in well-stoppered glass bottles, so as to be able to make, extemporaneously, a counter-irritating lotion of any requisite strength, according to the nature of the case requiring that application on extraordinary occasions; but for the ordinary purposes detailed in my work, it will be better to keep both a milder and a stronger ammoniated lotion ready prepared for use.

*The milder Ammoniated Lotion.*

Assuming the quantity of lotion desired to be divided into eight parts, then the proportions of the ingredients will stand thus:

A—four eighths;
R—three eighths;
C—one eighth.

*The stronger Ammoniated Lotion.*

If the quantity desired be also divided into eight parts, then the proportions of the ingredients run as follow:

A—five eighths;
R—two eighths;
C—one eighth.

Although the changes of proportion here may be deemed trifling, yet the strength of the lotion is such that I never employ it except in cases of apoplexy, and for the purpose of cauterization.

A and R are gradually mixed together. The mixture becomes opalescent and somewhat turbid, and a peculiar highly agreeable ethereal smell is given out, different from the individual odour of either ingredient, although the extreme pungency of the ammonia be still discernible.

Before the third ingredient is added, it is desirable to clear the previous mixture, by the addition of a small quantity of alcohol, and to set the whole in a cool place.

The lotion must always be kept in bottles with a glass stopper; and their whole virtue depends on the accurate distillation and preparation of the ingredients, as well as on the careful admixture of the latter.

*Lancet. October* 27, 1838.

